# Molecular characterization of a novel mycovirus in the cultivated mushroom, *Lentinula edodes*

**DOI:** 10.1186/1743-422X-9-60

**Published:** 2012-03-06

**Authors:** Yumi Magae

**Affiliations:** 1 Department of Applied Microbiology, Forestry and Forest Products Research Institute, Tsukuba, Ibaraki 305-8687, Japan

**Keywords:** Mycovirus, dsRNA, AFM, *Lentinula edodes*, Mushroom, NUDIX domain

## Abstract

**Background:**

In the 1970s, mycoviruses were identified that infected the edible mushroom *Lentinula edodes *(shiitake), but they were not regarded as causal agents for mushroom diseases. None of their genes has been sequenced. In this study, the dsRNA genome of a mycovirus recently found in a shiitake commercial strain was sequenced and its molecular structure was characterized.

**Methods:**

A cDNA library was constructed from a dsRNA purified from the fruiting body of *L. edodes*. The virus was tentatively named *L. edodes *mycovirus HKB (LeV). Based on the deduced RNA-dependent RNA polymerase (RdRp) sequence, phylogenetic analysis of LeV was conducted. Because no virion particles associated with the dsRNA were observed by electron microscopic observation, atomic force microscopy (AFM) observation was chosen for achieving molecular imaging of the virus.

**Results:**

The 11,282-bp genome of LeV was obtained. The genome encoded two open reading frames (ORFs). ORF1 coded for a hypothetical protein and ORF2 for a putative RdRp, respectively. In addition, a region coding for a NUDIX domain was present in ORF1. There was a 62-bp intergenic region between ORF1 and RdRp. Similarity with coat protein of mycoviruses was not found within the whole sequence. Based on phylogenetic analysis of the putative RdRp sequence, LeV grouped into a clade with dsRNA found in the basidiomycetes *Phlebiopsis gigantea *and *Helicobasidium mompa*. The clade was placed apart from the *Totiviridae *and *Chrysoviridae *families. As suggested from the genome sequence, AFM revealed that the structure of LeV was linear unencapsidated dsRNA.

**Conclusions:**

The results suggest that LeV represents a novel family of mycoviruses, found thus far only among the basidiomycetes.

## Background

In the 1970s, viruses that infect the cultivated mushroom *Lentinula edodes*, or shiitake, were extensively studied in Japan [1-3], and three morphologically distinct viruses were detected by electron microscopy [1,3]. However, unlike *La France *disease of the white button mushroom [4,5], mycoviruses have not been associated with shiitake diseases because these mycoviruses have commonly been found in healthy fruiting bodies [1,3]. In the USA, dsRNAs have also been observed in shiitake strains, but these appeared to be latent [6]. In the 1970s, shiitake cultivation was performed by inoculating mycelium spawn on oak logs; however, this labor-intensive method was gradually replaced by indoor cultivation using sawdust substrate supplemented with rice bran. Currently in Japan, about 75,016 t (82% of all shiitake) are produced indoors annually using bag cultures with a sawdust-based substrate [7]. In a bag culture, the shiitake mycelium is fully grown in the substrate until brown pigment is produced outside and the substrate becomes stiff. Complete browning of the exterior surface of the substrate is an important marker that normal fruiting bodies will develop in the following stage of cultivation. However, abnormal symptoms are occasionally observed in bag cultures, such as the growth of white or fluffy mycelia on the surface of substrate, inadequate or imperfect substrate browning [8], and malformations of the fruiting body. These symptoms often result in serious economic losses. Whether or not these abnormalities are associated with mycovirus is unknown. As a first step toward addressing that question, 46 shiitake isolates belonging to 11 commercial strains were examined for mycovirus infections [8]. As a result, dsRNA was found in two isolates; one showed imperfect browning and the other was asymptomatic. Agarose gel analysis showed that the isolate with imperfect browning contained several dsRNAs, but the asymptomatic isolate contained only a single dsRNA. In this study, the single dsRNA was tentatively designated as *Lentinula edodes *mycovirus HKB (LeV) and was sequenced.

## Methods

### dsRNA isolation

Three fruiting bodies were disrupted in 60 ml of 0.1 M phosphate buffer, and the virus fraction was precipitated with 10% PEG 8000 and 0.15 M NaCl, as described previously [9]. The PEG precipitate was suspended in TES (10 mM Tris-HCl, 1 mM EDTA, 0.15 M NaCl, pH 7.0), and total RNA was isolated using the QIAmp Viral RNA Mini Kit (Qiagen) according to the manufacturer's instructions. Then dsRNA was isolated from the viral RNA by DNase I (Promega) digestion for 30 min at 37°C, followed by S1 nuclease (TaKaRa) digestion. The resulting dsRNA was concentrated in nuclease-free water by filtration through Ultrafree 0.5 100 K centrifugal filters (Millipore) several times to remove degraded nucleic acids and salts. Finally, dsRNA was purified with the RNeasy Mini Elute Cleanup Kit (Qiagen).

### cDNA library construction and sequencing of dsRNA

The purified dsRNA served as a template for cDNA synthesis by random priming with the PrimeScript 1^st ^strand cDNA Synthesis Kit (TaKaRa) according to the standard protocol, except that the denaturing condition was changed from 65°C, 5 min to 98°C, 1 min. The resulting cDNAs were electrophoresed in an agarose gel, and cDNAs sized 1.5-2.0 kb were extracted. The cDNAs were blunt-ended with T4 DNA polymerase, ligated into pUC118/HincII/BAP (TaKaRa), and transformed into *E. coli *DH10B cells by electroporation (Gene Pulser, Bio-Rad). The resulting cloned DNA was sequenced with the BigDye Terminator v.3.1 Cycle Sequencing Kit (Applied Biosystems) with M13 forward and reverse primers. A contiguous region (contig) was assembled with Sequencher™ 4.6 (Gene Codes Corp).

### AFM microscopic observation

The PEG precipitate was suspended in 500 μl of TE and filtered through a 0.2-μm filter (Millipore), and 1 μl of the filtrate was diluted with 50 μl TE + 10 mM MgCl_2_. The sample was observed using atomic force microscopy (AFM) as previously described [10]. A total of 10 μl of the RNA solution was dropped onto freshly cleaved muscovite mica (1 × 1 cm), which after standing for several minutes was washed with distilled water. The sample was dried under a stream of nitrogen. Observations were performed on a MFP-3D (Asylum Research) in the tapping mode in air, using a silicon cantilever OMCL-AC240TS (Olympus). Fields of 2 μm × 2 μm were scanned at a frequency of < 2 Hz. To confirm that the AFM image observed was RNA, AFM imaging was also performed with PEG precipitate after RNaseA digestion. The length of the RNA molecule was measured using software developed by the Research Institute of Biomolecule Metrology Co., Ltd. (Japanese patent P2000-230823A).

### Phylogenetic analysis

An unrooted polygenetic tree was constructed with sequences retrieved by a PSI-BLAST [11] search (Table 1) using the neighbor-joining method with a bootstrap of 1000. The sequence identity was below 40%; thus, we used the Multiple Alignment using Fast Fourier Transform (MAFFT) program L9INS-I http://mafft.cbrc.jp/alignment/server/[12] for making the multiple alignments and constructing the phylogenetic tree. Sequences with bootstrap values above 70% were included in the tree and visualized on the web http://www.genome.jp/tools/mafft/

**Table 1:** RdRp sequences of viruses included in the phylogenetic tree

Virus	**Accession no**.	E-value	Identity	Similarity	References
*Lentinula edodes *mycovirus HKB LeVHKB	BAJ21197				This study

*Phlebiopsis gigantea *mycovirus dsRNA1 PgV1	CAJ34333	4e^-150^	383/1197 (32%)	580/1197 (48%)	[13]

*Helicobasidium mompa *V670 L2-dsRNA virus HmV670	AB275288	7e^-33^	26/65 (40%)	40/65 (62%)	unpublished

*Helminthosporium victoriae *145S virus HvV145S	AAM68953	1e^-15^	89/328 (27%)	141/328 (43%)	unpublished

*Verticillium chrysogenum virus *VcV	ADG21213	1e^-14^	99/406 (24%)	172/406 (42%)	unpublished

*Cryphonectria nitschkei chrysovirus *1 CnCV1	ACT79257	9e^-14^	83/318 (26%)	136/318 (43%)	[14]

*Penicillium chrysogenum *virus PcV	AAM95601	6e^-12^	109/455 (24%)	173/455 (38%)	[15]

Anthurium mosaic-associated virus AmV	ACU11563	5e^-11^	67/227 (30%)	101/227 (45%)	unpublished

Grapevine associated chrysovirus-1 GaCV1	ADO60926	5e^-09^	86/371 (23%)	138/371 (37%)	[16]

*Aspergillus fumigatus *chrysovirus AfCV	CAX48749	4e^-08^	102/455 (22%)	174/455 (38%)	[17]

*Circulifer tenellus *virus 1 CiTV1	ADK12924	5e-^07^	82/327 (25%)	135/327 (41%)	[18]

*Spissistilus festinus *virus 1 SpFV1	ADK12922	1e^-06^	84/338 (25%)	139/338 (41%)	[18]

*Saccharomyces cerevisiae *virus L-A ScVLA	AAA50508	2e^-05^	58/230 (25%)	100/230 (43%)	[19]

*Sphaeropsis sapinea *RNA virus 1 SsRV1	AAD11601	1e^-03^	48/202 (24%)	81/202 (41%)	[20]

E-values, identity, and similarity values were obtained from PSI-BLAST searches of the deduced amino acid data of RdRp sequences

Viruses that showed bootstrap values above 70% were included in the phylogenetic tree

## Results and discussion

### Genetic analysis

**Figure 1:**
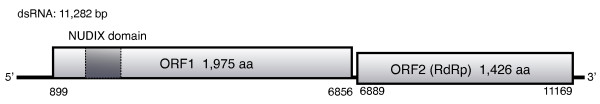
**Diagrammatic representation of genome organization of LeV**. The dsRNA of LeV encodes two ORFs. ORF1 is an unknown protein and has a region with high similarity with the NUDIX domain. ORF2 encodes the RNA-dependent RNA polymerase (RdRp).

A cDNA library was constructed from dsRNA purified from the fruiting body of *Lentinula edodes *strain HKB. An 11,282-bp cDNA contig containing two open reading frames (ORFs) was obtained (deposited under Accession No.AB429556) (Figure 1). ORF1 encoded a 218,428-Da protein composed of 1,975 amino acids, containing the conserved motif of a NUDIX domain (between 319aa and 451aa) (pfam00293, NUDIX, 1.56e^-04^) [21]. Viral coding NUDIX domain has been found only in *Poxviruses *[22]. LeV, as well as *Phlebiopsis gigantea *mycovirus dsRNA 1 is the first mycovirus described, that codes for NUDIX domain. ORF2 encoded a 162,240-Da protein composed of 1426 amino acids, containing the conserved motif of RNA-dependent RNA polymerase (RdRp) (pfam02123, RDRP_4, 4.48e^-22^). The deduced amino acid sequence of ORF1 showed significant similarity (3e^-83^) to hypothetical protein PgV-1_gp1 of *Phlebiopsis gigantea *mycovirus dsRNA 1 [13]. The deduced amino acid sequence of ORF2 showed similarity with members of *Totivirus *[23] and *Chrysovirus *[24]. The family *Totiviridae *includes all the viruses of fungi and protozoa that have virions and a single-component dsRNA genome. The family *Chrysovirus *includes dsRNA viruses that infect fungi or plants and have four genome components in isometric virions. Because LeV contained a single component dsRNA genome, it could be classified as a member of *Totivirus *[23]. But unlike *Totivirus*, there was no coding region for a coat protein within the LeV genome.

### AFM microscopic observation

**Figure 2:**
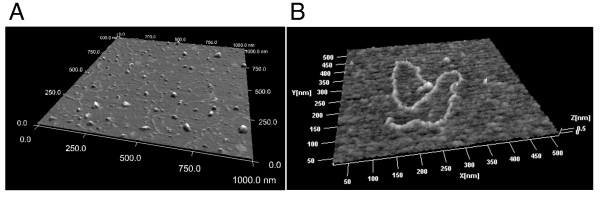
**AFM images of a LeV**. AFM images of PEG precipitate. (A) LeVs appear as numerous linear nucleic acids. (B) Image of a single LeV dsRNA.

**Figure 3:**
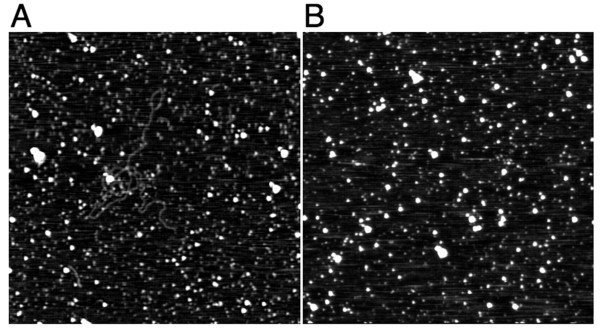
**AFM image of RNase-digested LeV**. (A) 2 μm × 2 μm AFM image of LeV. Length: 3,539 nm. (B) After exposure to RNaseA digestion.

No virion or vesicle particles were detected by electron microscopy observation of the PEG precipitate of LeV. Purification of the virus by sucrose density-gradient ultracentrifugation was unsuccessful. When the PEG precipitate was directly digested with RNase, the viral dsRNA could no longer be detected by agarose gel electrophoresis (data not shown). These experimental data suggested that the virus did not form a virion and therefore was vulnerable to excess purification. Because the method is nondestructive, AFM was chosen for further observation [25]. The PEG precipitate was suspended in TE and directly observed under AFM after being filtered through a 0.2-mm filter (Millipore). Numerous linear particles were observed (Figure 2A). The structure (Figure 2B) was very similar to the AFM images of dsRNA observed by Abels et al. and Vilfan et al. [26,27]. To confirm that these structures were RNA, PEG precipitate after RNase digestion was also observed by AFM (Figure 3B). After the RNase digestion, the linear materials were no longer present. Thus, LeV was revealed as a linear dsRNA. The length of the dsRNA was 3,539 nm (Figure 3A), and its molecular weight was estimated (based on 1 μm = ~3 kb dsRNA [27]) to be 11.8 kb. The estimated size was very close to that of the sequenced dsRNA (11,282 bp).

### Phylogenetic analysis

**Figure 4:**
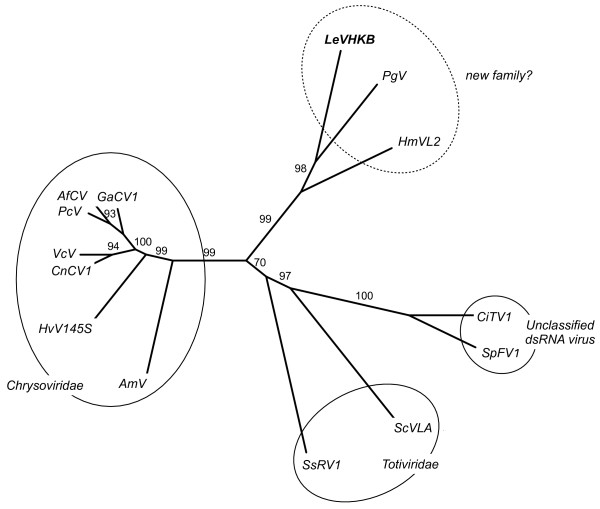
**Phylogenetic tree generated from the deduced amino acid sequences of RdRp**. The unrooted phylogenetic tree of the aligned sequences was constructed based on the neighbor-joining method using 1000 bootstrap replications. Numbers at the node indicate percentage of bootstrap supports. LeVHKB: *Lentinula edodes *mycovirus HKB; PgV: *Phlebiopsis gigantea *mycovirus dsRNA1; HmVL2: *Helicobasidium mompa *V670 L2-dsRNA virus (translated in this study from AB275288); CiTV1: *Circulifer tenellus *virus 1; SpFV1: *Spissistilus festinus *virus 1; ScVLA: *Saccharomyces cerevisiae *virus L-A; SsRV1: *Sphaeropsis sapinea *RNA virus 1; AmV: Anthurium mosaic-associated virus; HvV145S: *Helminthosporium victoriae *145S virus; CnCV1: *Cryphonectria nitschkei chrysovirus *1; VcV: *Verticillium chrysogenum virus*; PcV: *Penicillium chrysogenum *virus; AfCV: *Aspergillus fumigatus *chrysovirus; and GaCV1: Grapevine associated chrysovirus-1.

In the current classification of viruses, unencapsidated mycoviruses are placed in either the *Hypoviridae *[28,29] or *Endornaviridae *[30,31] families. However, the putative RdRp sequence of LeV showed no similarity to the RdRp sequences of *Hypovirus *or *Endornavirus*. An unrooted polygenetic tree was constructed with sequences retrieved by a PSI-BLAST [11] search (Table 1) using the neighbor-joining method with a bootstrap of 1000. Sequences with bootstrap values above 70% were included in the tree (Figure 4). This analysis grouped LeV with *Phlebiopsis gigantea *mycovirus dsRNA1 (E value; 4e^-150^) (CAJ34333) and *Helicobasidium mompa *V670 L2-dsRNA virus (translated in this study from AB275288) (E value; 7e^-33^) in a clade independent of *Totiviridae *and *Chrysoviridae *(Figure 4). Both *P. gigantea *and *H. mompa *are basidiomycetes. No virion particles associated with *P. gigantea *mycovirus dsRNA have been described [13]. Currently, whether *H. mompa *dsRNA forms virions or not is unknown. If *H. mompa *dsRNA was also unencapsidated, this would suggest that there is a novel family of mycoviruses so far found only in the basidiomycetes, having a monopartite dsRNA genome but do not package into virions.

### Hypothesis of unencapsidated virus in the basidiomycetes

*L. edodes *is a white-rot basidiomycete and *P. gigantea *is also a basidiomycete that causes white rot in conifer logs and stumps [32,33]. Often, *P. gigantea *is isolated from bark beetle, as is *Lentinula boryana*, a fungus belonging to the same family as *L. edodes *[34]. Virion structure is necessary for a virus to infect and exit from the host cell and gain a greater probability of propagation. Because bark beetles feed on basidiomycete fungi [34], the evolution of unencapsidated virus in white-rot basidiomycetes may have been achieved through their association with wood-feeding insects. Because their host can be efficiently transferred to new environments by bark beetles, the coat protein would no longer be necessary for virus propagation. Additionally, the conserved amino acids of putative RdRp molecules of unencapsidated viruses isolated from plant-feeding insects, such as the alfalfa hopper and beet leafhopper [18], are significantly similar to LeV (Figure 4) (Table 1). This fact also supports the hypothesis that unencapsidated species of mycovirus present in the basidiomycetes might be closely associated with insects.

## Conclusions

dsRNA found in a commercial strain of *Lentinula edodes *(shiitake) (designated as *L. edodes *mycovirus HKB; LeV) was sequenced. The 11.8-kb genome contained two ORFs. ORF1 coded for a hypothetical protein containing a NUDIX domain. Previously, viral NUDIX domain was found only in *Poxviruses*. LeV is the only mycovirus that codes NUDIX domain. ORF2 coded for RdRp with high similarity with *Totivirus *and *Chrysovirus*. The genome did not code for a coat protein, and AFM observation revealed LeV to be unencapsidated linear dsRNA. The results show that LeV is a novel mycovirus with a monopartite dsRNA genome that is closely related to *Totivirus*, but that it does not form a virion particle and might represent a new class of mycovirus.

## Competing interests

The author declares that they have no competing interests.
